# Plastome phylogenomics, biogeography, and clade diversification of *Paris* (Melanthiaceae)

**DOI:** 10.1186/s12870-019-2147-6

**Published:** 2019-12-05

**Authors:** Yunheng Ji, Lifang Yang, Mark W. Chase, Changkun Liu, Zhenyan Yang, Jin Yang, Jun-Bo Yang, Ting-Shuang Yi

**Affiliations:** 1 CAS Key Laboratory for Plant Diversity and Biogeography of East Asia, Kunming Institute of Botany, Chinese Academy of Sciences, Kunming, 650201 Yunnan China; 2 Yunnan Key Laboratory for Integrative Conservation of Plant Species with Extremely Small Population, Kunming Institute of Botany, Chinese Academy of Sciences, Kunming, 650201 Yunnan China; 3 Jodrell Laboratory, Royal Botanic Gardens, Kew, Richmond, TW9 3DS UK; 4 Germplasm Bank of Wild Species, Kunming Institute of Botany, Chinese Academy of Sciences, Kunming, 650201 Yunnan China

**Keywords:** Plastid phylogenomics, Biogeography, Radiative diversification, Cytonuclear discordance, Large genome size, Parideae, *Paris*, Melanthiaceae, Trilliaceae

## Abstract

**Background:**

*Paris* (Melanthiaceae) is an economically important but taxonomically difficult genus, which is unique in angiosperms because some species have extremely large nuclear genomes. Phylogenetic relationships within *Paris* have long been controversial. Based on complete plastomes and nuclear ribosomal DNA (nrDNA) sequences, this study aims to reconstruct a robust phylogenetic tree and explore historical biogeography and clade diversification in the genus.

**Results:**

All 29 species currently recognized in *Paris* were sampled. Whole plastomes and nrDNA sequences were generated by the genome skimming approach. Phylogenetic relationships were reconstructed using the maximum likelihood and Bayesian inference methods. Based on the phylogenetic framework and molecular dating, biogeographic scenarios and historical diversification of *Paris* were explored. Significant conflicts between plastid and nuclear datasets were identified, and the plastome tree is highly congruent with past interpretations of the morphology. Ancestral area reconstruction indicated that *Paris* may have originated in northeastern Asia and northern China, and has experienced multiple dispersal and vicariance events during its diversification. The rate of clade diversification has sharply accelerated since the Miocene/Pliocene boundary.

**Conclusions:**

Our results provide important insights for clarifying some of the long-standing taxonomic debates in *Paris*. Cytonuclear discordance may have been caused by ancient and recent hybridizations in the genus. The climatic and geological changes since the late Miocene, such as the intensification of Asian monsoon and the rapid uplift of Qinghai-Tibet Plateau, as well as the climatic fluctuations during the Pleistocene, played essential roles in driving range expansion and radiative diversification in *Paris*. Our findings challenge the theoretical prediction that large genome sizes may limit speciation.

## Background

*Paris* is a small genus that was once placed in Trilliaceae [1], but now in Melanthiaceae [2–6]. The genus comprises of ca. 29 species of understory perennial herbs that are continuously distributed across Eurasia [7–9]. With most species (24/29) occurring in China and Himalayas, *Paris* may have experienced significant species diversification in subtropical East Asia (21^°^–34^°^ N) [7, 9, 10]. Most species of this genus are much-valued traditional medicinal herbs in China and neighboring counties due to their various therapeutic properties [11–13]. Among them, the rhizomes of *Paris polyphylla* var. *chinensis* and *P. polyphylla* var. *yunnanensis* (*Rhizome Paridis*) have been used as traditional medicine for more than 2000 years in China [14]. To date, more than 40 commercial drugs and health products have been developed using *Rhizome Paridis* as raw materials [15], with ~ 1.5 billion USD per year in gross sales [16]. In addition, nearly all species with thick rhizomes are collected for medicinal purposes in Vietnam, Myanmar, Nepal, Bhutan, and India [13, 14].

*Paris* is morphologically distinctive in their single whorl of leaves (> 3) and solitary apical flower that is 4–15-merous. However, the rhizome, leaf, flower, stamens, ovary, fruit and seeds, which have been widely used to construct classifications, are highly divergent among species [7, 17]. Since the establishment of the genus by Linneaus [18], it has been subject to numerous critical revisions. Based on rhizome and fruit morphology, Franchet [19], who established the first infrageneric classification system of *Paris*, placed the species known at that time into two sections: *Euthyra* and *Paris*. Hara [17] described a third section, *Kinugasa*. Instead, Takhatajan [20] recognized these three sections as genera: *Paris s. s*. (= sect. *Paris*), *Daiswa* (= sect. *Euthyra*), and *Kinugasa* (= sect. *Kinugasa*). In the most comprehensive revision, Li [7] divided the genus into two subgenera, *Daiswa* and *Paris*, and eight sections, *Axiparis*, *Dunnianae*, *Euthyra*, *Fargesianae*, *Kinugasa*, *Marmoratae*, *Paris*, and *Thibeticae.* Based on molecular and morphological evidence, Ji et al. [21] suggested an updated classification of Li [7] by combining sections *Dunnianae*, *Fargesianae* and *Marmoratae* with *Euthyra*.

Several recent studies attempted to reconstruct phylogenetic relationships within *Paris* based on single or multiple DNA loci [21–24]. Due to insufficient sequence variation or limited taxon sampling, these studies did not provide satisfactory resolution or support for infrageneric relationships. As such, the absence of a solid phylogenetic scheme hinders the satisfactory resolution of the long-standing disagreements over classification of *Paris* and limits our understanding of the evolutionary and biogeographic history of this economically important genus.

Plant phylogenetics based on limited sequence regions often suffer from poor resolution and low support, particularly for clades in which rapid diversification or hybridization events have occurred [25–29]. Recently, next-generation sequencing, a technique capable of producing orders of magnitude more data than Sanger sequencing, has been increasingly used for phylogenetic reconstruction [30–35]. This has offered new approaches to resolve recalcitrant relationships in phylogenetically difficult taxa [36–43]. Huang et al. [44] and Yang et al. [45] attempted to apply plastid genomes (plastomes) to resolve phylogenetic relationships within *Pari*s. Although the plastome data greatly improve phylogenetic resolution and support, limited taxon sampling prevented them from building a robust overall view of the genus. It is, therefore, necessary to extend sampling size to cover all described sections and even all species, and to use markers with different inheritance patterns to comprehensively understand the evolutionary history of *Paris*.

The biparentally inherited but generally uniparental evolution via gene conversion of nuclear ribosomal DNA sequences (nrDNA) and the non-recombining, mostly maternally inherited plastomes contain a large number of evolutionarily informative variation suitable for phylogenetic analysis [46–50]. Genome skimming via shotgun sequencing of total genomic DNA at relatively low coverage is an efficient approach to recover entire plastomes and nrDNA [47]. Recently, genome skimming has been widely employed to reconstruct the evolutionary relationship at lower taxonomic levels and among closely related species [51–55], as well as to investigate reticulate evolution in diverse plant clades [52, 56–58]. In this study, we generated plastome and nrDNA sequences from all currently recognized *Paris* species using genome skimming method. Based on phylogenomic analyses, we aimed to (1) clarify evolutionary relationships within *Paris*; and (2) explore biogeographic scenarios and historical diversification for the genus.

## Results

### Illumina sequencing and assembly

Low coverage genome sequencing generated per sample 8.57–35.73 million paired-end clean reads (150 bp) (Additional file 1: Table S1). Of these, 7.25 × 10^4^ to 2.10 × 10^6^ and 4.81 × 10^3^ to 4.59 × 10^4^ were mapped to the reference plastome and nuclear nrDNA, respectively. Based on these data, we assembled complete plastomes and nrDNA for all samples, with the average sequencing depth ranging from 68.72–1998.57 times and 75.15–1136.47 times, respectively.

The de novo assembly produced 33 *Paris* plastomes, which exhibited a typical quadripartite structure, with the size varying from 156,139–158,643 bp (Additional file 2: Figure S1). *Paris* plastomes are conserved in gene content and arrangement. All plastomes contain 114 genes, including 80 protein-coding genes, 30 tRNA genes, and four plastid rRNA genes (Additional file 3: Table S2). Alignment of the plastomes yielded a matrix of 166,726 positions, in which we identified 5899 variable sites (3.53%) and 3225 (1.93%) were parsimony informative (Table 1). Also, our de novo nrDNA assembly entirely covered 18S, ITS1, 5.8S, ITS2 and 26S regions. The sequence length of *Paris* nrDNA ranged from 5840 to 5859 bp. Alignment of the nrDNA sequences produced 443 variable sites (7.56%), of which 264 (4.50%) were parsimony informative (Table 1).

**Table 1:** Comparison of sequence characteristics of the aligned plastome and nrDNA datasets in *Paris*

Dataset	Aligned length (bp)	Variable sites (divergence)	Parsimony informative sites (divergence)
Complete plastome	166,726	5899 (3.53%)	3225 (1.93%)
nrDNA	5862	443 (7.56%)	264 (4.50%)

### Phylogenetic relationships

**Fig. 1:**
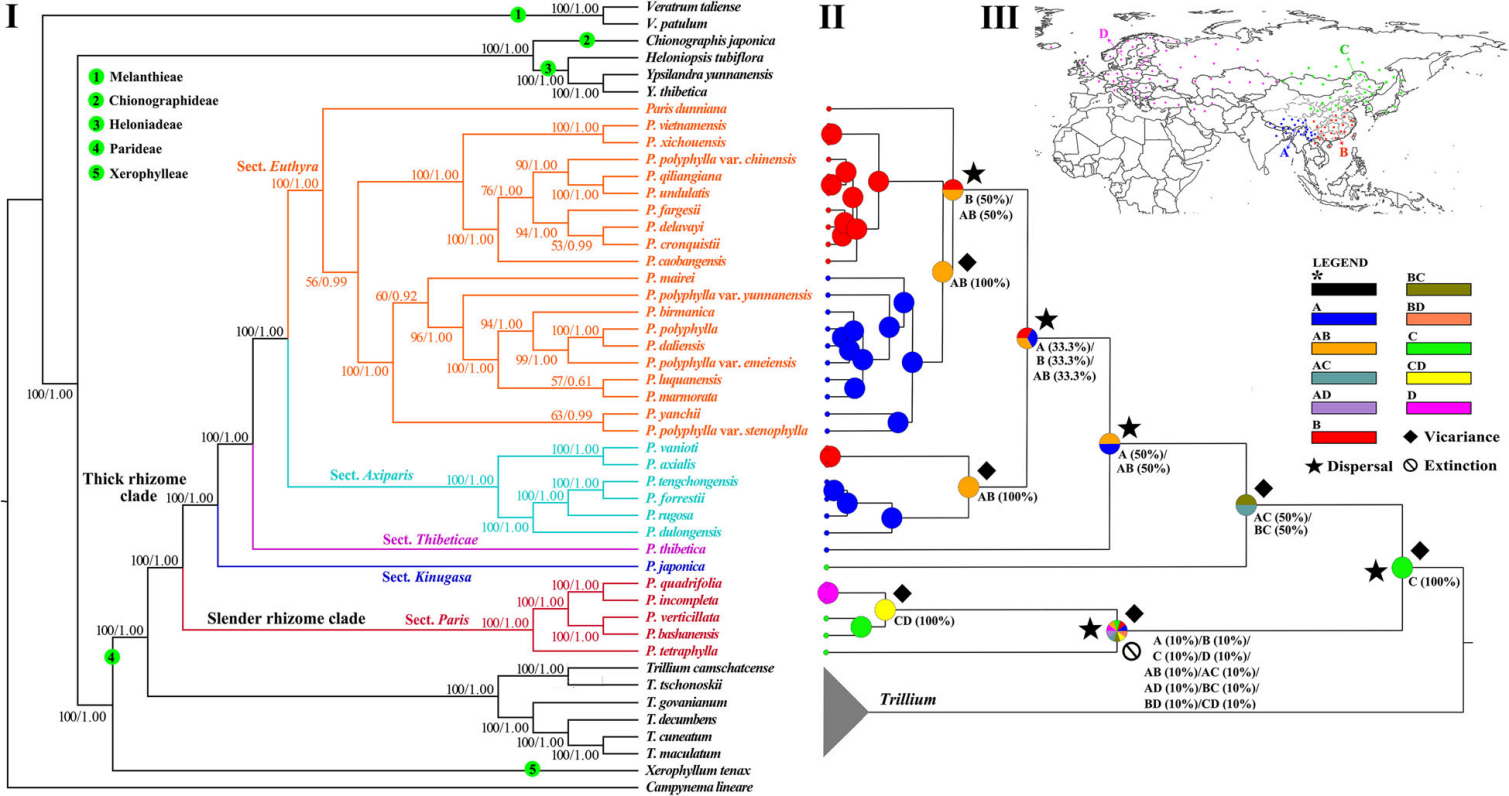
Phylogenetic relationships and ancestral areas reconstruction of *Paris.* (**I**) Phylogenetic tree based on plastome DNA sequences. Numbers above branches indicate maximum likelihood bootstrap percentages (BP) and Bayesian posterior probabilities (PP). (**II**) Reconstruction of ancestral area of *Paris* using S-DIVA analysis inferred from plastid tree. (**III**) *Paris* species assigned to four areas based on their current distributions: A. southwestern China and Himalayas, B. eastern, central, southern China and northern Indochina, C. northeastern Asia and northern China, D. Europe and Caucasus

The standard maximum likelihood (ML) and Bayesian inference (BI) analyses of complete plastomes generated identical tree topologies (Fig. 1). Five highly supported clades (bootstrap percentage, BP = 100; posterior probability, PP = 1.00) within Melanthiaceae were resolved, which correspond to the five tribes recognized by Zomlefer [3]. Their relationships are congruent with those of previous studies [3, 5, 6, 44, 59]. The monophyly of *Paris* was strongly supported (BP = 100, PP = 1.00), which was sister to *Trillium* (BP = 100, PP = 1.00). Within *Paris*, five well-supported clades corresponding to the five sections circumscribed by Ji et al. [21] were recovered. Our results support the successive divergence of the *P.* sections *Paris*, *Kinugasa*, *Thibeticae*, *Axiparis* and *Euthyra*. Most relationships obtained high support except a few terminal species relationships. For instance, the relationship between *P. luquanensis* and *P. marmorata* received weak branch support (BP = 57, PP = 0.67) in both ML and BI trees.

**Fig. 2:**
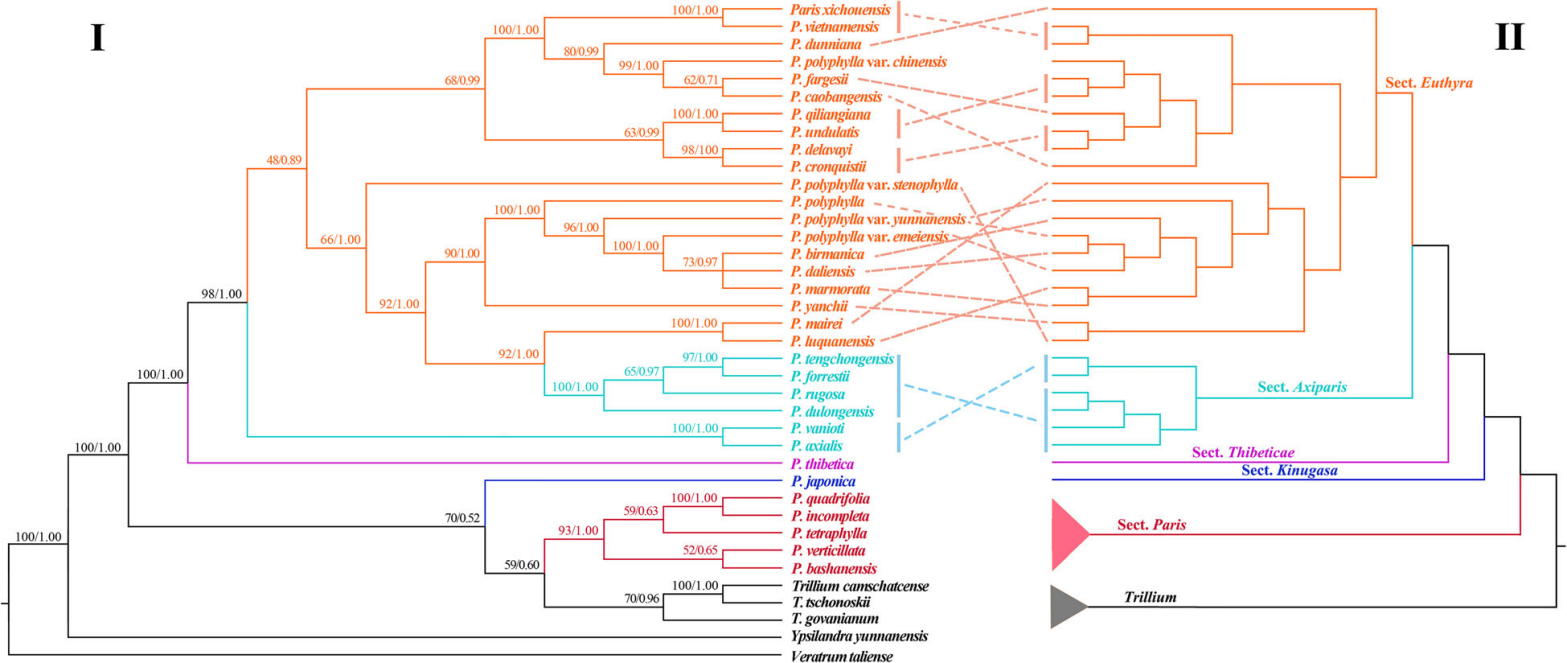
Comparison of tree topologies recovered from analyses of nuclear ribosomal DNA sequences (**I**) and plastome DNA sequences (**II**). Numbers above branches indicated maximum likelihood bootstrap percentages (BP) and Bayesian posterior probabilities (PP)

The incongruence length difference (ILD) test revealed significant discordance (*p* < 0.001) between the nrDNA and plastome datasets. The phylogenetic analysis of nrDNA sequences (Fig. 2) divided tribe Paridae (*Paris* and *Trillium*) species into two clades (BP = 100, PP = 1.00). Within the first clade (BP = 70, PP = 0.50), section *Paris* is sister to *Trillium* (BP = 59, PP = 0.60) and this pair is sister to section *Kinugasa*. Within the second clade (BP = 100, PP = 1.00), section *Thibeticae* is sister to all species of sections *Axiparis* and *Euthyra*, which are non-monophyletic. In comparing nuclear and plastid topologies, we observed three instances of cytonuclear discordance at different taxonomic levels (Fig. 2). The first is the non-monophyly of *Paris* in the nuclear dataset. The second is monophyly of sections *Axiparis* and *Euthyra*: they are paraphyletic in nrDNA tree but monophyletic in the plastid tree. The third instance of cytonuclear discordance concerns the interspecific relationships within section *Euthyra* (Fig. 2).

### Ancestral area reconstruction, molecular dating, and historical diversification

The Statistical-Dispersal Vicariance Analysis (S-DIVA) (Fig. 1) reconstructed northeastern Asia and northern China (C) as the ancestral area for the most recent common ancestor (MRCA) of *Paris*. It may have undergone a westward or southward dispersal into southwestern China and Himalayas or eastern, central, southern China and northern Indochina [AC (0.50/BC (0.50)] to evolve the MRCA of thick rhizome clade. Then, a vicariance was inferred to split section *Kinugasa* (Japanese Islands) from remaining taxa (sections *Thibeticae*, *Axiparis*, and *Euthyra*, subtropical East Asia); within the latter, three dispersal and two vicariance events were inferred. Although the S-DIVA analysis failed to reconstruct the ancestral area of the section *Paris*, a dispersal, an extinction, and two vicariance events were inferred in the clade.

**Fig. 3:**
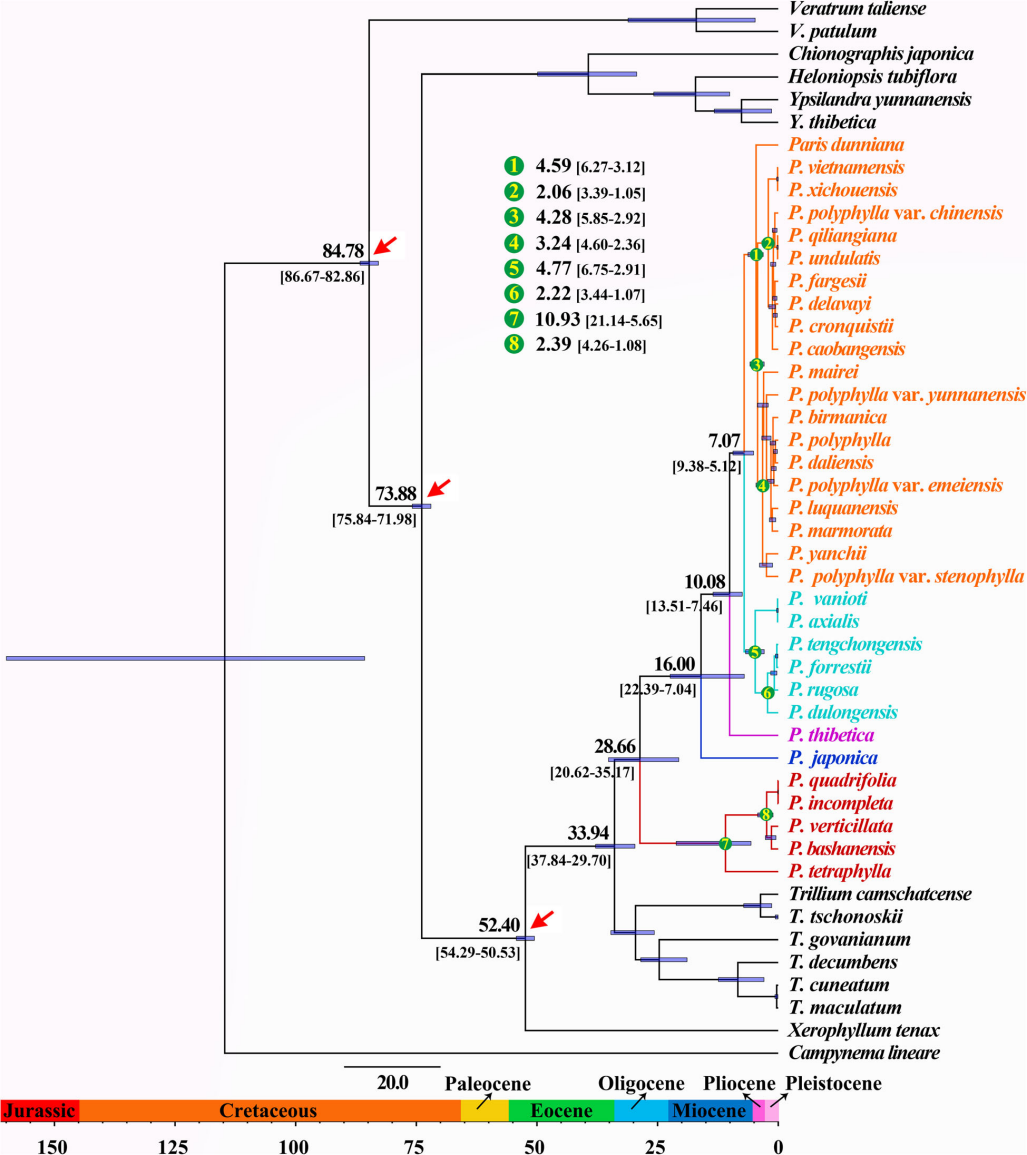
Divergence time estimation based on plastome DNA sequences. Numbers above/under the tree branches represented mean divergent ages and 95% confidence interval of each node. Red arrows indicate the calibration points for the molecular dating. Divergence time and the timeline are indicated in million years (Mya)

The BEAST analyses (Fig. 3) indicated that the divergence between the sister genera, *Paris* and *Trillium*, occurred at 33.94 Mya (95% HPD: 37.84–29.70 Mya). Within *Paris*, the thick and slender rhizome clades diverged from their MRCA at 28.66 Mya (95% HPD: 35.17–20.62 Mya), around the early Oligocene. Diversification within the thick rhizome clade commenced 16.00 Mya (95% HPD: 22.39–7.04 Mya), around the early Miocene, leading to the divergence of the monotypic section *Kinugasa* from the remaining thick-rhizome taxa. Subsequently, the monotypic section *Thibeticae* diverged from the MRCA of sections *Axparis* and *Euthyra* at 10.08 Mya (95% HPD: 13.51–7.46 Mya), in late Miocene. The split of sections *Axparis* and *Euthyra* was dated at 7.07 Mya (95% HPD: 9.38–5.12 Mya), around the Miocene/Pliocene boundary. Additionally, the diversification of sections *Paris*, *Axiparis*, and *Euthyra* occurred at 10.93 Mya (95% HPD: 21.14–5.65 Mya), 4.77 Mya (95% HPD: 6.75–2.91 Mya), and 4.59 Mya (95% HPD: 6.27–3.12 Mya), respectively.

**Fig. 4:**
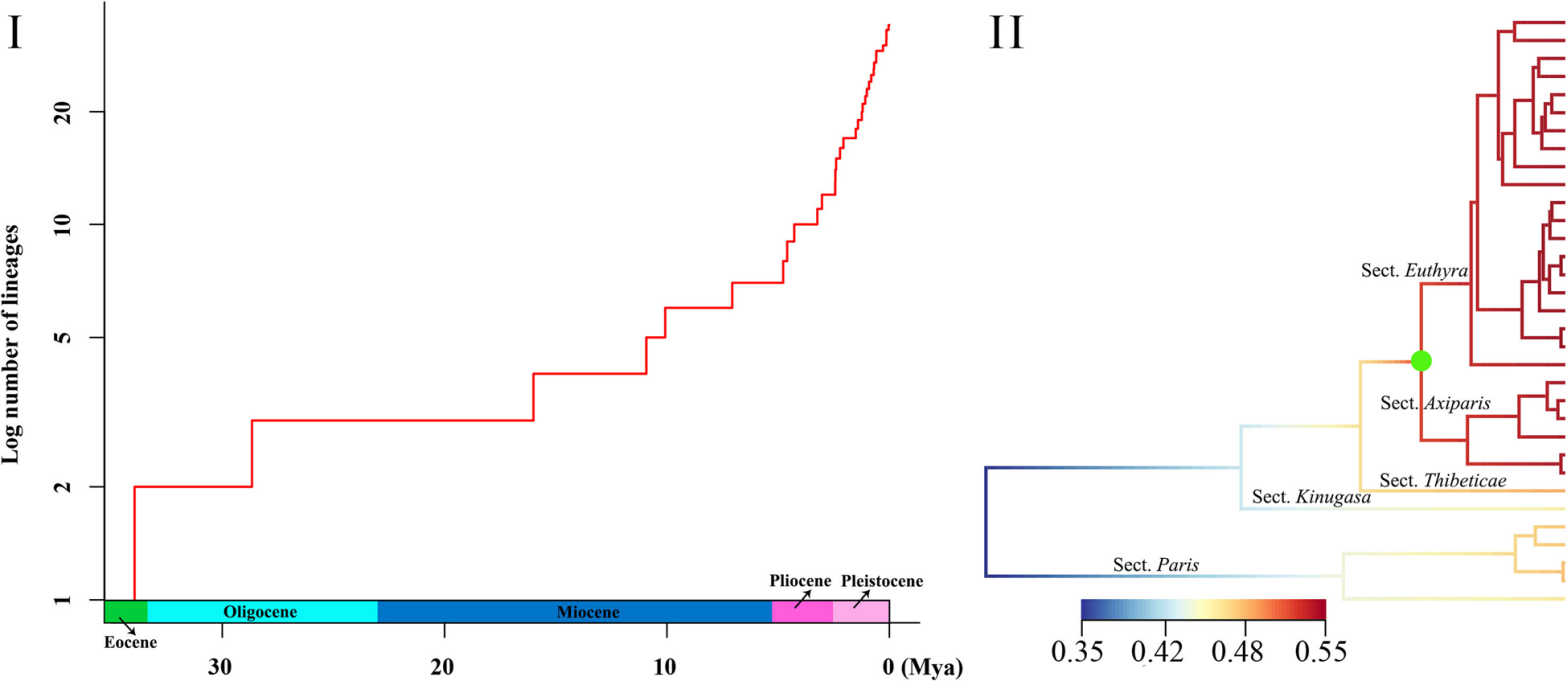
(**I**) Result of lineage through time (LTT) plots (A) analyses. Mya = million years ago. (**II**) Rate shift with the highest posterior probability inferred in Bayesian Analysis of Macroevolutionary Mixtures (BAMM) on the time-calibrated maximum clade credibility tree from BEAST. A shift is predicted and shown with green circle. Colors on the branch represent the mean of the posterior density of net diversification rate (speciation rate minus extinction rate)

The semi-logarithmic lineage through time (LTT) plots analysis (Fig. 4) suggested that the origin of *Paris* was followed by a relatively stable diversification rate, which, however, sharply increased around Miocene/Pliocene boundary. This upward trend was maintained during the Pliocene and the Pleistocene. The Bayesian Analysis of Macroevolutionary Mixtures (BAMM) detected a rate shift in net species diversification in *Paris*, which occurred with the divergence between sect. *Euthyra* and *Axiparis* (Fig. 4).

## Discussion

### Phylogenetic inferences and taxonomic implications

Previous phylogenetic analyses based on a small number of DNA loci or limited taxon sampling failed to robustly reconstruct the backbone of the *Paris* tree [21–24, 44, 45]. Including all currently recognized species, the plastome analysis fully resolved interspecific relationships of *Paris* with strong support at most nodes. Our study further confirms that phylogenetic analysis based on more DNA loci with greatly increased number of phylogenetically informative characters can significantly improve resolution at low taxonomic levels [36, 43, 52, 53, 60].

**Fig. 5:**
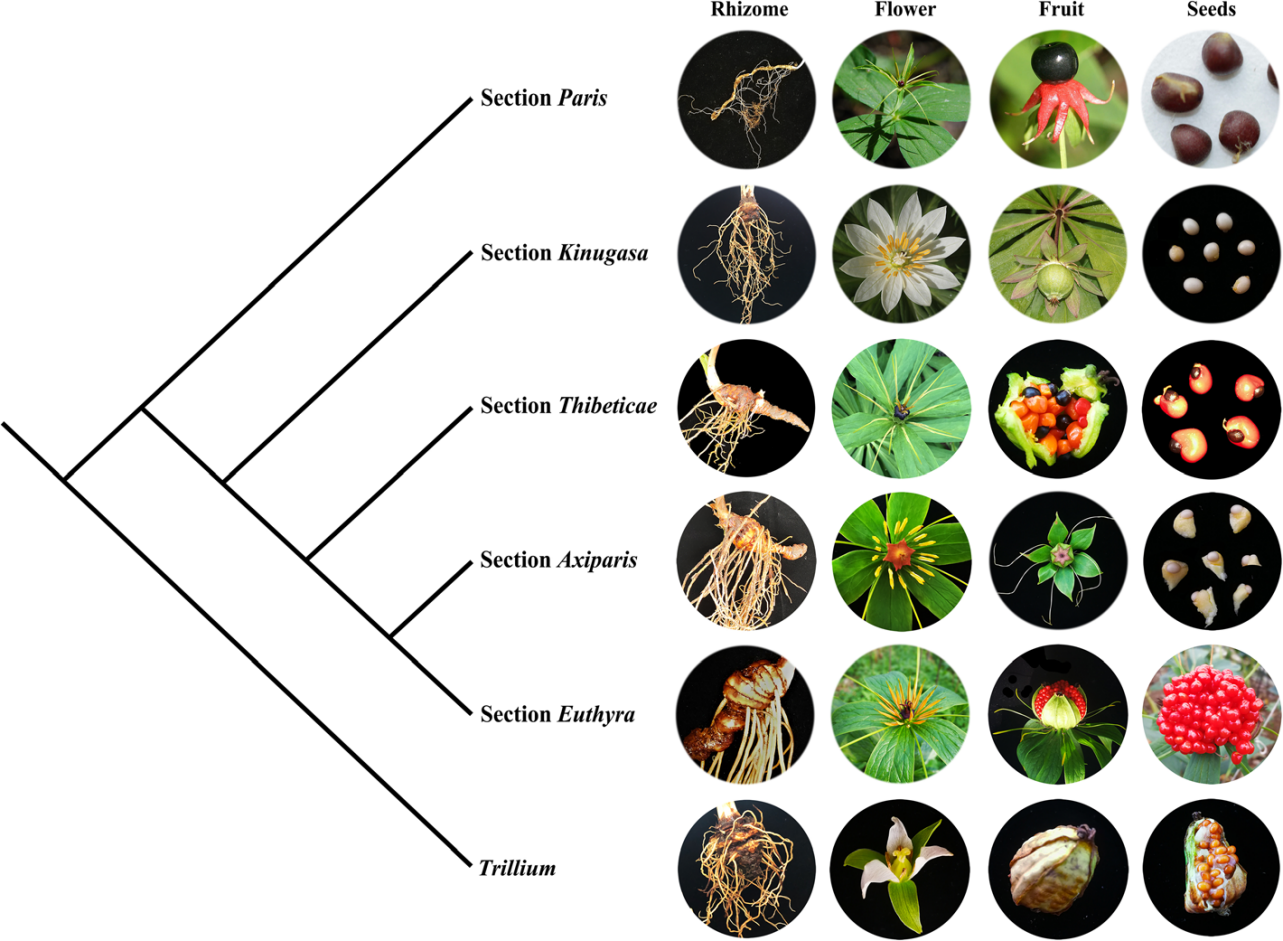
Comparison of morphological features among five *Paris* sections and *Trillium*

The plastome-based phylogenies strongly support the monophyly of *Paris* and recovered five strongly supported major clades that correspond to the previously proposed sections by Ji et al. [21]. Within *Paris*, successive divergence along the spine of the tree of sections *Paris*, *Kinugasa*, *Axiparis*, *Thibeticae*, *Axiparis*, and *Euthyra* was inferred. This divergence pattern can be supported by some morphological characters (Fig. 5). Briefly, the slender rhizome and round more berry-like fruit distinguish section *Paris* from the rest of the sections. Nevertheless, seeds without an enveloping sarcotesta (or aril, presumably a plesiomorphic character) separate sections *Paris* and *Kinugasa* from the rest. Although species of the thick rhizome clade (including sections *Kinugasa*, *Thibeticae*, *Axiparis*, and *Euthyra*) commonly have angular fruits, section *Kinugasa* is distinctive among these in possessing showy white sepals. The third diverging section *Thibetica* is similar to *Euthyra* in having dehiscent capsules, but its seed morphology (with an incomplete aril) is analogous to that of *Axiparis*. Therefore, the plastid tree is highly congruent with the morphologically based classification of Ji et al. [21].

The plastid tree provides valuable insights for resolving the long-standing disagreements in classification of *Paris*. All *Paris* species share the morphological synapomorphies of flowers and leaves being 4–15-merous compared with the trimerous condition of *Trillium* (Fig. 5), and monophyly of both genera was fully supported, making it reasonable to recognize *Paris* as a single genus [7, 17, 21] rather than dividing it into three genera (*Daiswa*, *Kinugasa*, and *Paris s. s.*) [20]. Also, the new tree further supports the taxonomic treatment of Ji et al. [21] by combining sections *Dunnianae*, *Fargesianae* and *Marmoratae* with *Euthyra*. Given its economic importance, resolution of the long-standing taxonomic disputes will be conducive to exploration and protection of *Paris* species.

The current taxonomy of *P. polyphylla* with five varieties is not supported by either the plastid or nrDNA results. The varieties should be probably recognized as distinct species, but it is also likely, given the cytonuclear discordance observed for these accessions (Fig. 2) that hybridization may be involved in their origins. Further study of this group with more appropriate population genetic and cytological techniques is warranted.

### Cytonuclear discordance

Similar to the previous study of Ji et al. [21], we detected discordance between the nrDNA and plastid trees for *Paris* at both deep and shallow nodes. Cytonuclear incongruence is a fairly common phenomenon in plant phylogenetics [25, 56, 61–68]. In most cases, the nuclear tree is more congruent with morphological taxonomy [43, 56, 61, 62, 64, 67–70], and such incongruence can be mainly attributed to incomplete sorting of cytoplasmic polymorphisms or introgression of the cytoplasmic genome from one species into the nuclear background of another by hybridization [25, 63, 68, 71, 72]. However, in our study the plastid tree of *Paris* is largely consistent with morphological evidence, suggesting nrDNA introgression without cytoplasmic gene flow could be responsible for the discordance detected [68, 71–74].

Given that the discordance observed in *Paris* was likely due to phenomena affecting the nrDNA tree, which involved rapid gene conversion of one parental copy [75–80], the phylogenetic relationships recovered by this dataset may not be representative of that obtained with other parts of the nuclear genome not subject to gene conversion. Nonetheless, these results may provide useful information about past hybridization events that otherwise may not be the majority pattern in the nuclear genome. Failure to recover *Paris* as a monophyletic in the nrDNA tree (Fig. 2) suggests there could have been hybridization between section *Paris* and *Trillium* after section *Paris* split form the rest of the genus. It is noteworthy that the largest eukaryotic genome, that *P. japonica* [81, 82], was supposed to be an allo-octaploid between *Paris* and *Trillium* according to previous cytological investigations [83]. This hybrid hypothesis is supported by its position as sister to section *Paris* and *Trillium* in the nrDNA tree. Likewise, the non-monophyly of sections *Axiparis* and *Euthyra* observed in the nrDNA tree (Fig. 2) can also be attributed to ancient hybridization. The sister relationship of *P. luquanensis*/*P. mairei* (section *Euthyra*) and the clade of four species of section *Axiparis* (*P. dulongensis*, *P. forrestii*, *P. rugosa* and *P. tengchongensis*) suggest that hybridization could have occurred between the ancestors of these taxa. Additionally, extensive discordance among species of section *Euthyra* (Fig. 2) supports the conclusions of the previous study that natural hybridization between species of section *Euthyra* is likely if the pollinators are the same, but little is known about this aspect of the biology of *Paris*. Experimental manual outcrossing has been effective between most of these species [84]. Interspecific hybridization is the likely cause of the cytonuclear discordance observed between species in the section. Additionally, as mentioned above, there is discordance for the positions of the varieties of *P. polyphylla*, suggesting that hybridization may also have played a role in their origins.

It is notable that the cytonuclear discordance detected in this study merely reflects conflict between plastomes and nrDNA datasets, which are substantially two linkage groups of plastid and ribosomal genes and only represent at best two large single-locus DNA regions. Whether there is nuclear genome-wide and plastome discordance is a gigantic leap with such a limited dataset. To further verify this, large numbers of unlinked nuclear loci generated by restricted site-associated DNA sequencing (RAD-seq) or even whole nuclear genome sequencing are likely to be required.

### Biogeography and lineage diversification

Because strong cytonuclear discordance was detected in *Paris* and the plastid tree agrees well with morphologically based classification, we address the biogeography and historical diversification of *Paris* based on the plastid dataset. The S-DIVA analysis recovered northeastern Asia and northern China as the ancestral area of *Paris*. Associated with a dispersal and a vicariance event (Fig. 1), the crown node of *Paris* was dated at 28.66 Mya (Fig. 3), in the early Oligocene, when the global climatic deterioration [85] led to the expansion of vegetation adapted to drier and colder climates in large parts of Eurasia [86]. Therefore, early divergence of *Paris* may have been driven by these events. Also, S-DIVA analysis revealed that the divergence of the Japanese endemic species, *P. japonica* and *P. tetraphylla* was triggered by two independent vicariance events (Fig. 1). Their divergence times of 16.00 Mya (*P. japonica*) and 10.93 Mya (*P. tetraphylla*), in the Miocene corresponds to the opening of the Japan Sea, which separated the Japan islands from the continental East Asia [87].

In the thick rhizome clade, the S-DIVA analysis inferred three dispersal events (Fig. 1), which were dated at 10.08, 7.07 and 4.59 Mya (Fig. 3), respectively. Neocene climatic change might play an essential role in triggering these events. The onset of the Asian monsoon around the Oligocene/Miocene transition created a connection between forests from low to high latitudes of East Asia [88]. The enhancement of Asian summer monsoon since the late Miocene established a humid climate in subtropical East Asia [89–91], and caused a significant expansion of forests in East Asia [88, 92]. These climatic and environmental shifts would create favorable habitats that facilitated the dispersal and divergence of sections *Thibeticae*, *Axiparis* and *Euthyra* in subtropical East Asia. Also, along with the expansion of forests in high latitudes of East Asia, the MRCA of *P. quadrifolia* and *P. incompleta* may have migrated into Europe.

Both LTT and BAMM analyses revealed that clade diversification within *Paris* abruptly accelerated around the Miocene/Pliocene boundary, which could be driven by the further strengthening of monsoonal climate in the summer and the initiation of the two distinct monsoon regimes that have gradually become established in subtropical East Asia since the late Miocene [88, 92, 93]. From then on, eastern, central and southern China and northern Indochina have been primarily governed by Pacific monsoon, whereas southwestern China and the Himalayas have been mainly affected by Indian monsoon [92–95]. The S-DIVA analysis (Fig. 1) and molecular dating (Fig. 3) showed that vicariance events occurred independently in sections *Axiparis* (4.77 Mya) and *Euthyra* (4.28 Mya) in the two regions mentioned above. This implies profound ecological heterogeneity resulting from climate differentiation may have driven significant allopatric speciation in the two regions [96–98]. In addition, the LTT and BAMM analyses (Fig. 4) revealed that most extensive divergence in *Paris*, which was responsible for appearance of more than half of the extant taxa, took place in the Pliocene and the Pleistocene. It is believed that the Qinghai-Tibet Plateau (QTP) rose dramatically from the Late Miocene (ca. 10~8 Ma) to the early Pliocene (ca. 3.6 Ma) [99, 100], which dramatically modified global climate [94, 101] and thereby profoundly influenced biological processes, such as species range expansion/contraction and vicariance, in East Asia [102]. During the Pleistocene, there were at least four major glaciations in East Asia [103], and these probably created significant isolation and diverse habitats in East Asia [104, 105]. Such complex geological, ecological, and environmental heterogeneity is expected to have driven diversification of a wide spectrum of plant clade [104, 106–109] and would also have triggered vicariance and facilitated a species radiative in *Paris*.

A negative correlation between genus-level diversity and the genus-average genome size was observed in plants [110, 111]. Knight et al. [111] proposed the large genome constraint hypothesis, which states that plant taxa with large size genomes diversify more slowly. Subsequently, Suda et al. [112] found that many island clades of Macaronesian angiosperms that underwent adaptive radiations have small genome sizes, and assumed that rapid diversification is more likely to happen in angiosperms with small genomes size. It is noteworthy that *Paris* is fairly distinctive in angiosperms in possessing large genomes. The minimum documented genome size in the genus (*P. verticillata*, 1C = 30.52 Gb) is much larger than the mean genome size (1C = 5.7 Gb) of angiosperms [113, 114]. Moreover, the known largest eukaryotic genome, that of *P. japonica*, 1 C = 148.88 Gb, belongs to *Paris* [81, 82]. In this study, we found that *Paris* may have undergone a species radiation since the Miocene/Pliocene boundary (Fig. 4), which is not consistent with prediction that large genome size could limit speciation [111, 112]. It also has to be admitted that although there is sharp rise in the lineage diversification, the total number of species involved is not large in comparison to other radiations, for instance, *Dianthus* in the Mediterannean [115], and *Aizoaceae* in South Africa [116]. The generality of the large genome constraint hypothesis needs to be further evaluated, although the increased lineage diversification detected here in *Paris* does not pose a major contradiction to it.

## Conclusions

This study represents a comprehensive phylogenetic investigation of *Paris*, an economically important but taxonomically difficult genus, by sampling all currently recognized species in the genus. The analyses of complete plastome and nrDNA sequences reconstructed a robust phylogeny, and provided implications for clarifying some of the long-standing taxonomic debates in *Paris*. We also identified significant conflicts between plastid and nuclear datasets. This cytonuclear discordance observed in *Paris* may have been caused by ancient and recent hybridizations. Ancestral area reconstruction indicated that *Paris* may have originated in northeastern Asia and northern China, and has experienced multiple dispersal and vicariance events during its diversification. Based on phylogenetic framework and molecular dating, we propose that the climatic and geological changes since the Miocene played essential roles in triggering range formation and clade diversification in *Paris*. Our findings provide important insights for elucidating the evolutionary history of *Paris*, and will be conducive to exploration and protection of *Paris* species.

## Methods

### Plant sampling, DNA extraction and Illumina sequencing

We sampled 33 accessions to represent all 29 species and five varieties recognized by Li [7] and those described since then [9, 84, 117–119]. The original sources of the plant materials used in this study and voucher information are presented in Additional file 4: Table S3. The voucher specimens were identified by Dr. Yunheng Ji. Genomic DNA was extracted from ca. 20 mg silica gel dried leaves using the CTAB (cetyltrimethylammonium bromide) method [120]. Approximately 5 μg of purified genomic DNA was sheared by sonication. Paired-end libraries with an average insert size 350 bp were prepared using a TruSeq DNA Sample Prep Kit (Illumina, Inc., USA) according to the manufacturer’s protocol. The libraries were paired-end sequenced on the Illumina HiSeq 2000 platform. Raw reads were filtered to remove adaptors and low quality reads using NGS QC Toolkit [121], by setting the cut-off value for percentage of read length to 80 and PHRED quality scores at 30.

### Assembly and gene annotation

The complete plastome sequence of *Paris quadrifolia* (GenBank Accession: KM067394) was used as the reference for assembling the newly sequenced *Paris* plastomes. The plastid contigs were organized according to the references and connected with overlapping terminal sequences to yield the complete plastomes in Bowtie v2.2.6 [122] using the default parameters. Plastomes were annotated with the Dual Organellar Genome Annotator database [123]. Start and stop codons and intron/exon boundaries for protein-coding genes were checked manually. Annotated tRNA genes were further verified by tRNAscan-SE 1.21 [124] with the default parameters. The boundary of the large-single copy (LSC), small-single copy (SSC), and inverted-repeat (IR) regions for each plastome were visually examined and manually adjusted according to those of the reference plastome in Geneious V10.2 [125].

For nrDNA sequence assembly, we first excluded all plastid-like reads. Based on remaining reads, de novo assemblies were performed using the complete nrDNA sequence (including 26S, 18S and 5.8S ribosomal RNA genes and the internal transcribed spacers) of *Lillium tsingtauense* (GenBank Accession: KM117263) as reference. The external transcribed spacers in *Paris* species possesses too many repeat sequences and inversions that may make the assembly inaccurate, we therefore did not assemble the region. Contigs mapping to reference nrDNA were assembled using the processes described above. The nuclear ribosomal RNA genes and their boundaries with ITS regions were annotated and defined by comparison with the reference in Geneious V10.2 [125].

### Phylogenetic analysis

To investigate phylogenetic placement of *Paris* within Melanthiaceae, 15 other plastomes representing the five tribes recognized in Melanthiaceae (Additional file 5: Table S4) were integrated with the 33 newly sequenced *Paris* plastomes in the final analysis. Furthermore, 33 *Paris* nrDNA and five rDNA sequences from *Veratrum* (1 accession), *Ypsilandra* (1 accession) and *Trillium* (3 accessions) of Melanthiaceae were incorporated into a nuclear dataset (Additional file 4: Table S3). *Campynema lineare* and *Veratrum taliense* were used to root the plastid and nuclear trees, respectively, according to previous studies [3, 5, 6, 59]. Alignment of plastid and nrDNA sequences were performed using MAFFT [126] integrated in Geneious v.10.2 [125], and manually edited if necessary. The most appropriated model of sequence substitution for plastomes (GTR + G) and nrDNA sequences (GTR + I + G) was selected using Modeltest v3.7 [127] with the Akaike information criterion [128]. We considered the whole plastome as a single inherited unit. Next, we confirmed the same model for both the small and large single copy regions and the inverted repeats using PartitionFinder v. 2.1.1 [129]. Conflict between plastid and nuclear datasets was examined using the incongruence length difference (ILD) test [130] implemented in PAUP* 4.0b10 [131] for 1000 replicates.

Phylogenetic analyses were carried out using both ML and BI methods. ML analyses were conducted using RAxML-HPC BlackBox v8.1.24 [132] with 1000 replicates of rapid bootstrapping. The BI analyses were performed using MRBAYES v.3.1.2 [133]. Runs for each dataset began with a random starting tree for one million generations with sampling at every 100 generations. An initial 25% of the sampled trees were discarded. The posterior probability values were determined from the remaining trees. Stationarity was considered to be reached when the average standard deviation of the split frequencies was < 0.01.

### Molecular dating and diversification rate estimate

It is notable that no fossils have been identified for Melanthiaceae and its close relatives. A previous study that used 17 fossils across the monocots and major clades of angiosperms suggested that the crown age of Melanthiaceae was approximately 84.8 Mya, while the clades Parideae-Xerophyllideae and Chionographideae-Heloniadeae diverged approximately 74 Mya, and the tribes Parideae and Xerophyllideae split approximately 52.3 Mya [134]. We used these times to calibrate the phylogenetic tree (Fig. 3). Molecular dating was performed in BEAST v.2.4.7 [135]. The BEAST analyses were run under the uncorrelated lognormal relaxed clock approach with a Yule tree prior. Markov Chain Monte Carlo chains were run for 10,000,000 were run with sampling every 1000 generations. The stationarity of the chains and convergence of BEAST analyses was monitored by Tracer v. 1.5.

The diversification rate change over time was inferred using the semi-logarithmic lineage through time (LTT) plot approach. The consensus chronogram inferred from the results of molecular dating was computed by APE v.5.3 [136] within an R environment [137]. We further examined potential shifts in net diversification rate in *Paris* based on the time calibrated maximum clade credibility tree (with the highest posterior probability) from BEAST using the Bayesian Analysis of Macroevolutionary Mixtures (BAMM) [138].

### Ancestral area reconstruction

For biogeographic reconstructions, *Paris* species were assigned to four areas based on their current distributions: A) southwestern China and Himalayas, B) eastern, central, southern China and northern Indochina, C) northeastern Asia and northern China, and D) Europe and the Caucasus. Ancestral distributions of *Paris* were reconstructed by statistical dispersal-vicariance analysis (S-DIVA) [139] implemented in RASP 4.0 [140]. The condensed tree and 4000 post burn-in Bayesian trees from BI analysis were used as input trees. The random tree was defined as 1000 and other parameters were set to their defaults.

## Supplementary information

**Additional file 1: Table S1.** Summary of Illumina sequencing.

**Additional file 2: Figure S1.** Plastome map of *Paris* species.

**Additional file 3: Table S2.** Gene content of *Paris* plastomes.

**Additional file 4: Table S3.** Samples used in the study, with voucher and source information, and Genbank accessions.

**Additional file 5: Table S4.** Sequences downloaded from Genbank.

## Data Availability

The sequences generated in this study are available at GenBank (accession numbers are presented in Table S3). The manually adjusted alignment of sequences are deposited in Treebase (**http://purl.org/phylo/treebase/phylows/study/TB2:S25166**).

## Abbreviations

BI: Bayesian inference
bp: Base pair
BP: Bootstrap percentage
BS: Bootstrap
CTAB: Cetyl trimethylammonium bromide
DNA: Deoxyribonucleic acid
HPD: Highest posterior density
ILD: Incongruence length difference
IR: Inverted repeat
ITS: Internal transcribed spacer of nuclear ribosomal DNA
KUN: Herbarium of Kunming Institute of Botany, Chinese Academy of Sciences
LSC: Large single-copy
LTT: Semi-logarithmic lineage through time
MCMC: Markov Chain Monte Carlo
ML: Maximum likelihood
MRCA: Most recent common ancestor
Mya: Million years ago
nrDNA: Nuclear ribosomal DNA sequences
PP: Posterior probability
QTP: Qinghai-Tibet Plateau
rRNA: Ribosomal RNA
S-DIVA: The statistical-dispersal vicariance analysis
SSC: Small single copy
tRNA: Transfer RNA
USD: United States dollar
