# Supplementary material for: Plastome phylogenomics, biogeography, and clade diversification of Paris (Melanthiaceae)
Source: BMC Plant Biol. 2019 Dec 5;19:543. doi: 10.1186/s12870-019-2147-6 (PMC6896732; doi:10.1186/s12870-019-2147-6)
Supplement: Supplementary file 1 — Additional file 1: Table S1. Summary of Illumina sequencing. [file 12870_2019_2147_MOESM1_ESM.docx]

**Table S1. Summary of Illumina sequencing.**

| Species | No. of total reads | Plastome | | |  | rDNA | | |
| --- | --- | --- | --- | --- | --- | --- | --- | --- |
|  |  | No. of maped reads | Sequence length (bp) | Coverage (×) |  | No. of maped reads | Sequence length (bp) | Coverage (×) |
| *Paris dunniana* | 21,458,050 | 492,700 | 158,256 | 467.091 |  | 14,776 | 5,857 | 376.811 |
| *Paris polyphylla* var. *chinensis* | 18,722,992 | 508,212 | 158,406 | 481.797 |  | 8,233 | 5,855 | 209.954 |
| *Paris fargesii* | 14,077,220 | 359,355 | 158,643 | 340.677 |  | 5,610 | 5,855 | 143.064 |
| *Paris delavayi* | 26,865,254 | 77,315 | 158,575 | 73.296 |  | 29,116 | 5,856 | 742.503 |
| *Paris caobangensis* | 17,125,486 | 466,961 | 158,256 | 442.690 |  | 9,524 | 5,857 | 242.877 |
| *Paris mairei* | 19,963,868 | 391,942 | 157,753 | 371.570 |  | 13,926 | 5,840 | 355.134 |
| *Paris polyphylla* var. *yunnanensis* | 24,043,696 | 526,568 | 157,874 | 499.199 |  | 11,187 | 5,856 | 285.286 |
| *Paris birmanica* | 20,647,760 | 593,449 | 157,857 | 562.603 |  | 11,174 | 5,856 | 284.954 |
| *Paris marmorata* | 12,466,238 | 85,380 | 157,740 | 80.942 |  | 22,446 | 5,856 | 572.407 |
| *Paris yanchii* | 24,313,490 | 547,701 | 157,918 | 519.233 |  | 19,029 | 5,855 | 485.269 |
| *Paris tengchongensis* | 8,574,500 | 72,494 | 157,150 | 68.726 |  | 10,800 | 5,855 | 275.417 |
| *Paris rugosa* | 21,815,948 | 452,693 | 157,239 | 429.163 |  | 14,329 | 5,855 | 365.411 |
| *Paris thibetica* | 24,994,840 | 545,760 | 157,389 | 517.393 |  | 21,264 | 5,856 | 542.265 |
| *Paris vietnamensis* | 31,809,342 | 588,783 | 158,101 | 558.180 |  | 40,858 | 5,856 | 1041.942 |
| *Paris cronquistii* var. *xichouensis* | 23,895,584 | 1,271,474 | 158,225 | 1205.387 |  | 17,401 | 5,856 | 443.752 |
| *Paris qiliangiana* | 20,197,880 | 679,904 | 158,354 | 644.565 |  | 23,795 | 5,856 | 606.809 |
| *Paris undulates* | 21,082,716 | 862,479 | 158,286 | 817.650 |  | 24,208 | 5,856 | 617.341 |
| *Paris polyphylla* | 31,809,342 | 588,781 | 158,130 | 558.178 |  | 40,859 | 5,856 | 1041.967 |
| *Paris daliensis* | 35,730,218 | 1,082,892 | 158,118 | 1026.607 |  | 45,944 | 5,856 | 1171.642 |
| *Paris luquanensis* | 21,222,976 | 1,563,752 | 157,901 | 1482.473 |  | 7,528 | 5,840 | 191.976 |
| *Paris polyphylla* var. *stenophylla* | 20,953,708 | 352,947 | 157,833 | 334.602 |  | 25,232 | 5,856 | 643.455 |
| *Paris forrestii* | 19,953,306 | 576,609 | 157,198 | 546.639 |  | 11,817 | 5,855 | 301.352 |
| *Paris japonica* | 25,745,358 | 408,200 | 155,957 | 386.983 |  | 6,643 | 5,849 | 169.407 |
| *Paris quadrifolia* | 26,621,402 | 2,103,930 | 157,350 | 1998.578 |  | 6,978 | 5,857 | 177.950 |
| *Paris verticillata* | 21,974,100 | 306,370 | 157,946 | 291.029 |  | 6,596 | 5,856 | 168.208 |
| *Paris bashanensis* | 24,083,578 | 166,862 | 157,320 | 158.507 |  | 4,808 | 5,856 | 122.611 |
| *Paris tetraphylla* | 24,970,044 | 115,564 | 156,567 | 109.777 |  | 8,659 | 5,859 | 220.818 |
| *Paris polyphylla* var. *emeiensis* | 16,105,910 | 325,184 | 158,406 | 308.282 |  | 14,929 | 5,856 | 380.712 |
| *Paris cronquistii* | 19,216,610 | 289,832 | 158,586 | 274.767 |  | 7,759 | 5,856 | 197.866 |
| *Paris vanioti* | 15,904,162 | 106,838 | 156,846 | 101.285 |  | 8,768 | 5,854 | 223.597 |
| *Paris axialis* | 16,167,930 | 155,331 | 156,821 | 147.257 |  | 8,406 | 5,854 | 214.366 |
| *Paris dulongensis* | 22,155,212 | 362,234 | 157,342 | 343.406 |  | 44,565 | 5,855 | 1136.476 |
| *Paris incompleta* | 14,973,694 | 14,594,660 | 157,610 | 1382.911 |  | 2,952 | 5,857 | 75.159 |
