# Supplementary figures and images for: Plastome phylogenomics, biogeography, and clade diversification of Paris (Melanthiaceae)
Source: BMC Plant Biol. 2019 Dec 5;19:543. doi: 10.1186/s12870-019-2147-6 (PMC6896732; doi:10.1186/s12870-019-2147-6)

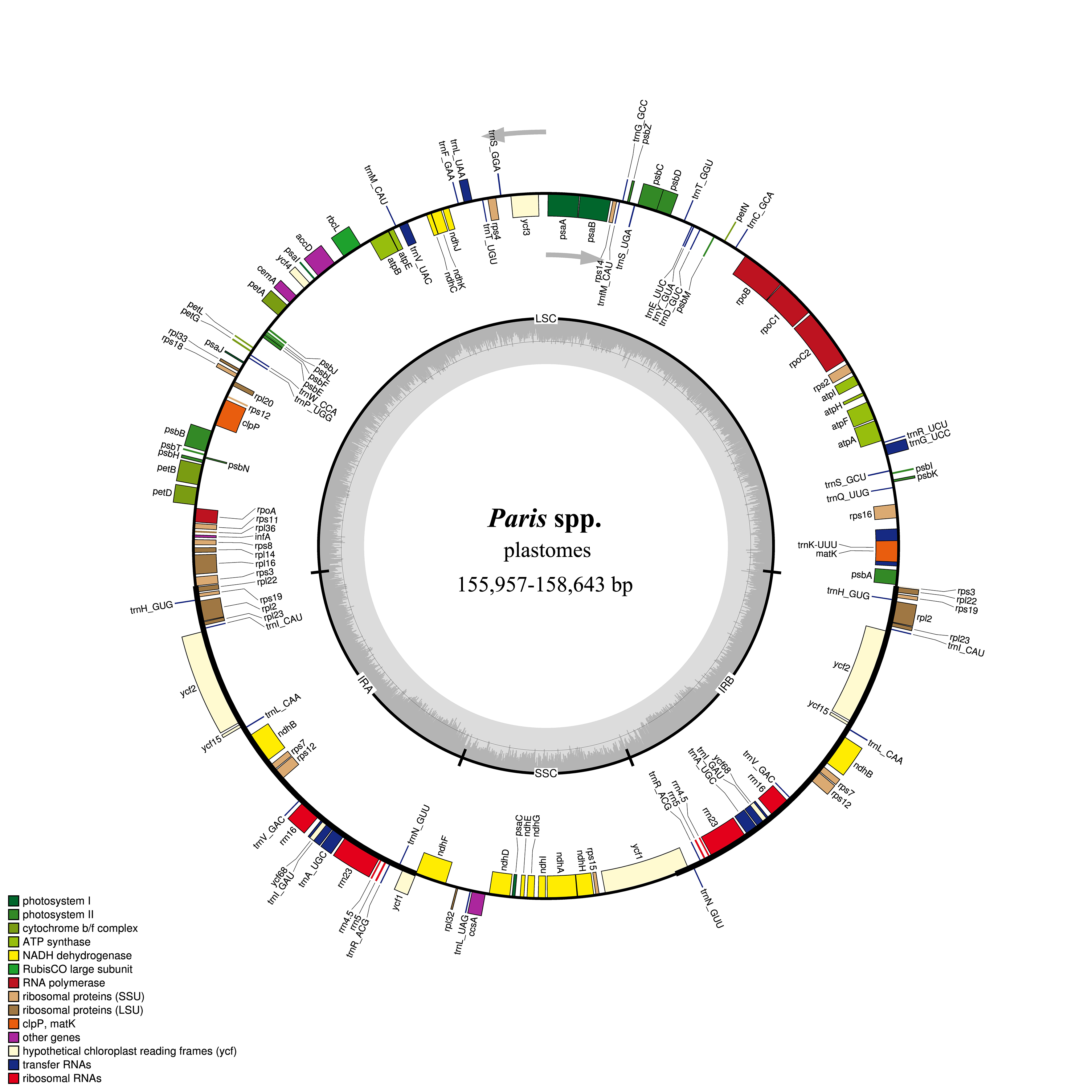

Supplement: Supplementary file 2 — Additional file 2: Figure S1. Plastome map of Paris species. [file 12870_2019_2147_MOESM2_ESM.jpg]
