# Supplementary material for: Plastome phylogenomics, biogeography, and clade diversification of Paris (Melanthiaceae)
Source: BMC Plant Biol. 2019 Dec 5;19:543. doi: 10.1186/s12870-019-2147-6 (PMC6896732; doi:10.1186/s12870-019-2147-6)
Supplement: Supplementary file 4 — Additional file 4: Table S3. Samples used in the study, with voucher and source information, and Genbank accessions. [file 12870_2019_2147_MOESM4_ESM.docx]

Table S3. Samples used in the study, with voucher and source information, and GenBank accessions.

| Samples | Locality | Vouncher | GenBank Accession | |
| --- | --- | --- | --- | --- |
|  |  |  | Plastome | Ribosomal DNA |
| *Paris dunniana* Lévl. | Wuzhishan, Hainan, China | HYL‒34 | MN125592 | MN174888 |
| *P. mairei* Lévl. | Lijiang, Yun nan, China | Y. Ji 20160392 | MN125598 | MN174891 |
| *P. birmanica* (Takht.) H. Li | Introduced from northern Myanmar | Y. Ji 2016433 | MN125571 | MN174895 |
| *P. marmorata* Stearn | Shangri-La, Yunnan, China | Y. Ji 2016503 | MN125597 | MN174869 |
| *P. daliensis* H. Li et V. G. Soukup | Lanping, Yunnan, China | Y. Ji et al. 1859 | MN125579 | MN174885 |
| *P. polyphylla* var. *emeiensis* H. X. Yin, H. Zhang et D. Xue | Ya’an, Sichuan, China | Y. Ji et al. s. n. | MN125583 | MN174904 |
| *P. polyphylla* Smith | Jianchuan, Yunnan, China | L. Yang 1848 | MN125589 | MN174883 |
| *P. luquanensis* H. Li | Luquan, Yunnan, China | Y. Ji et al. 1865 | MN125573 | MN174886 |
| *P. polyphylla* var. *chinensis* (Franch.) Hara | Enshi, Hubei, China | Y. Ji 2016407 | MN125588 | MN174892 |
| *P. caobangensis* Y. H. Ji, H. Li et Z. K. Zhou | Guangnan, Yunnan, China | Y. Ji 2016618 | MN125593 | MN174871 |
| *P. fargesii* Franch. | Sangzhi , Hunan, China | Y. Ji 2016414 | MN125595 | MN174893 |
| *P. cronquistii* (Takht.) H. Li | Wanyuan, Sichuan, China | Y. Ji et al. s. n. | MN125574 | MN174903 |
| *P. undulates* H. Li et V. G. Soukup | Emeishan, Sichuan, China | Y. Ji et al. 1832 | MN125586 | MN174879 |
| *P. qiliangiana* H. Li, J. Yang et Y. H. Wang | Xuanhan, Sichuan, China | Y. Ji et al. 1834 | MN125576 | MN174880 |
| *P. delavayi* Franch. | Daguan, Yunnan, China | Y. Ji 2016519 | MN125581 | MN174870 |
| *P. xichouensis* H. Li. | Malipo, Yunnan, China | Y. Ji et al. 1820 | MN125585 | MN174894 |
| *P. vietnamensis* (Takht.) H. Li | Jinping, Yunnan, China | Y. Ji et al. 1810 | MN125575 | MN174878 |
| *P. polyphylla* var. *stenophylla* Franch. | Luding, Sichuan, China | Y. Ji 2018051 | MN125590 | MN174884 |
| *P. yanchii* H. Li, L. -G. Lei et Y. -M. Yang | Jianchuan, Yunnan, China | Y. Ji 2016482 | MN125582 | MN174868 |
| *P. polyphylla* var. *yunnanensis* (Ftanch.) Hand. -Mazz | Wuding, Yunnan, China | Y. Ji 626 | MN125587 | MN174873 |
| *P. tengchongensis* Y. H. Ji, C. J. Yang et Y. L. Huang | Tengchong, Yunnan, China | Y. Ji 361 | MN125584 | MN174889 |
| *P. forrestii* (Takht.) H. Li | Gongshan, Yunnan, China | G. Zhou s. n. | MN125565 | MN174877 |
| *P. rugosa* H. Li et S. Kurita | Changning, Yunnan, China | Y. Ji 2016525 | MN125570 | MN174872 |
| *P. dulongensis* H. Li et S. Kurita | Gongshan, Yunnan, China | H. Li 57 | MN125566 | MN174887 |
| *P. vanioti*Lévl. | Xinning, Hunan, China | H. Li 052 | MN125567 | MN174901 |
| *P. axialis* H. Li | Zhaotong, Yunnan, China | Y. Ji s. n. | MN125591 | MN174902 |
| *P. thibetica* Franch. | Tengchong, Yunnan, China | Y. Ji 20160387 | MN125569 | MN174890 |
| *P. japonica* (Franch. et Sav.) Franch. | Cult. in Royal Botanical Garden Edinburgh | 1974145813B | MH796668 | MN174876 |
| *P. bashanensis* Wang et Tang | Pengzhou, Sichuan,China | H. Liang and X. Zhang 1332 | MN125580 | MN174874 |
| *P. verticillata*M. Bieb. | Chicheng, Hebei, China | B. Liu 3490 | MH796669 | MN174881 |
| *P. quadrifolia* L. | Netherlands | P. Bruggeman s. n. | MN125594 | MN174882 |
| *P. incompleta*M. Bieb. | Georgia | Z. Zhou and H. Sun s. n. | MN125572 | MN174898 |
| *P. tetraphylla* A. Gray | Japan | K. Inoue and T. Kubo 1609 | MN125596 | MN174875 |
| *Trillium camschatcense* Ker Gawl. | Panshi, Jilin, China | H. An 090 | MN125568 | MN174899 |
| *T. tschonoskii* Maxim. | Shangri-La, Yunnan, China | YLF‒95 | MN125577 | MN174897 |
| *T. govanianum* Wall. ex D. Don | Dingri, Tibet, China | H. Li and S. Chen 391 | MH796670 | MN174867 |
| *Ypsilandra yunnanensis* W. W. Sm. et Jeffrey | Fugong, Yunnan, China | GLGS Expedition 33923 | MH796672 | MN174896 |
| *Veratrum taliense* O. Loes. | Gongshan, Yunnan, China | YLF‒107 | MN125578 | MN174900 |
