# Supplementary material for: Plastome phylogenomics, biogeography, and clade diversification of Paris (Melanthiaceae)
Source: BMC Plant Biol. 2019 Dec 5;19:543. doi: 10.1186/s12870-019-2147-6 (PMC6896732; doi:10.1186/s12870-019-2147-6)
Supplement: Supplementary file 5 — Additional file 5: Table S4. Sequences downloaded from Genbank. [file 12870_2019_2147_MOESM5_ESM.docx]

Table S4. Sequences downloaded from Genebank.

| Species | Order | Family | GenBank Accession |
| --- | --- | --- | --- |
| *Trillium cuneatum* | Liliales | [Melanthiaceae](https://en.wikipedia.org/wiki/Melanthiaceae) | KR135077 |
| *Trillium maculatum* | Liliales | [Melanthiaceae](https://en.wikipedia.org/wiki/Melanthiaceae) | KR780075 |
| *Trillium decumbens* | Liliales | [Melanthiaceae](https://en.wikipedia.org/wiki/Melanthiaceae) | KR534612 |
| *Ypsilandra thibetica* | Liliales | [Melanthiaceae](https://en.wikipedia.org/wiki/Melanthiaceae) | MH796671 |
| *Chionographis japonica* | Liliales | [Melanthiaceae](https://en.wikipedia.org/wiki/Melanthiaceae) | KF951065 |
| *Heloniopsis tubiflora* | Liliales | [Melanthiaceae](https://en.wikipedia.org/wiki/Melanthiaceae) | KM078036 |
| *Xerophyllum tenax* | Liliales | [Melanthiaceae](https://en.wikipedia.org/wiki/Melanthiaceae) | KM078035 |
| *Veratrum patulum* | Liliales | [Melanthiaceae](https://en.wikipedia.org/wiki/Melanthiaceae) | KF437397 |
| *Campynema lineare* | Liliales | Campynemataceae | KP462881 |
